# Automated Detection of Cervical Carotid Artery Calcifications in Cone Beam Computed Tomographic Images Using Deep Convolutional Neural Networks

**DOI:** 10.3390/diagnostics12102537

**Published:** 2022-10-19

**Authors:** Maryam Ajami, Pavani Tripathi, Haibin Ling, Mina Mahdian

**Affiliations:** 1 School of Dental Medicine, Stony Brook University, Stony Brook, NY 11794, USA; 2 Department of Computer Sciences, Stony Brook University, Stony Brook, NY 11794, USA; 3 Department of Prosthodontics and Digital Technology, School of Dental Medicine, Stony Brook University, Stony Brook, NY 11794, USA

**Keywords:** carotid arteries, carotid stenosis, cone beam computed tomography, artificial intelligence, deep learning

## Abstract

The aim of this study was to determine if a convolutional neural network (CNN) can be trained to automatically detect and localize cervical carotid artery calcifications (CACs) in CBCT. A total of 56 CBCT studies (15,257 axial slices) were utilized to train, validate, and test the deep learning model. The study comprised of two steps: Step 1: Localizing axial slices that are below the C2–C3 disc space. For this step the openly available Inception V3 architecture was trained on the ImageNet dataset of real-world images, and retrained on 40 CBCT studies. Step 2: Detecting CACs in slices from step 1. For this step, two methods were implemented; Method A: Segmentation neural network trained using small patches at random coordinates of the original axial slices; Method B: Segmentation neural network trained using two larger patches at fixed coordinates of the original axial slices with an improved loss function to account for class imbalance. Our approach resulted in 94.2% sensitivity and 96.5% specificity. The mean intersection over union metric for Method A was 76.26% and Method B improved this metric to 82.51%. The proposed CNN model shows the feasibility of deep learning in the detection and localization of CAC in CBCT images.

## 1. Introduction

Dental cone beam computed tomography (CBCT) is an advanced tomographic imaging modality for visualization of maxillofacial structures in three dimensions. The use of CBCT is growing rapidly in various dental fields such as oral surgery, implant dentistry, orthodontics, and endodontics. With the increase in the number of CBCT scans in recent years, there is growing concern about significant incidental findings going unnoticed [1]. Cervical carotid artery calcification is a potentially significant incidental finding in the soft tissue of the neck that is captured in dental CBCT scans with a prevalence rate of 5.7–11.6% and up to 30% in older populations [2]. There is a correlation between cervical carotid artery calcification and ischemic cardiovascular disease [3,4]. This finding can be overlooked by dentists with limited familiarity with soft tissue anatomy due to minimal soft tissue contrast in dental CBCT images. Moreover, numerous soft tissue structures in the vicinity of the carotid artery bifurcation can calcify which are of non-clinical significance. Thus, accurate and early detection of cervical vascular calcifications and distinguishing them from other non-life-threatening calcifications in this region is imperative for prevention and proper management of the disease [2,5,6,7,8].

Artificial intelligence (AI) is a technology that is used in many areas of our lives with a significant impact on healthcare [9]. Deep learning is a subset of AI that uses a mathematical construct called a convolutional neural network (CNN) that is modeled after interconnected neurons in a brain [10,11,12]. Instead of relying on an expert to identify predefined features as in machine learning and traditional computer vision techniques, CNNs are programmed to automatically extract and learn features from a large volume of training data with feedback to optimize the outcome. Deep learning methods have surpassed human-level performance in tasks such as image classification, object detection, localization, and image segmentation [13,14,15,16].

Healthcare research in AI has increased dramatically over the years with an increase of 45.15% since 2014 [17]. In dentistry, AI can be used in diagnosis, treatment planning and prognosis of disease, especially in radiology, with emphasis in image segmentation and disease identification [18,19]. Several systematic reviews have investigated the applications of AI in dental radiology reporting a vast majority on 2D imaging and for applications such as tooth identification, detection of dental caries, periodontal disease and periapical pathology [20]. While in recent years, the landscape of dental AI literature has gradually expanded to include 3D imaging, further research is needed to investigate the feasibility of this technology for various diagnostic tasks and to improve the performance of the algorithms for employment in the daily practice of dentists. Several studies have applied computer-aided techniques for CAC detection in panoramic imaging, ultrasonography and computed tomography [21,22,23,24,25]. However, to our knowledge, there are no studies to apply CNN for the detection of CACs in CBCT images. Therefore, the aim of the present study was to develop a deep learning technique that can be used to detect and localize cervical carotid artery calcifications in the dental CBCT modality with an acceptable performance.

## 2. Materials and Methods

The study was reviewed and approved by the Institutional Review Board (IRB) at Stony Brook University (1384974-3). The dataset used for this study was retrieved from the existing CBCT scans acquired at the Stony Brook University School of Dental Medicine Dental Care Center between 2009 and 2019. The scans were acquired for implant treatment planning using the I-CAT Digital imaging machine (Imaging Sciences International, Hatfield, PA, USA) at 120 kVp, 5 mA, 12 Seconds and voxel size of 0.3 mm and the raw Digital Imaging and Communications in Medicine (DICOM) files were deidentified using the Anatomage Invivo 5 software (Anatomage Inc. Santa Clara, CA, USA). The existence of cervical carotid artery calcification at the bifurcation, approximately at C3–C4 vertebra, was confirmed by the radiology report by a board-certified Oral and Maxillofacial Radiologist in the patient’s chart serving as ground truth. 40 CBCT scans (11,252 axial slices) were used for training the neural networks, and 16 CBCT scans (4005 axial slices) for testing it. In each set, half of the CBCT scans were positive for carotid artery calcification. Scans with calcified triticeous cartilage and superior cornu of thyroid cartilage were excluded from the study.

We divided the problem into two separate steps and trained a different neural network for each step:

1. Finding axial slices that are below the C2–C3 disc space (Classification of slices)
2. Finding carotid artery calcifications in slices from step 1.

### 2.1. Step 1: Classification of Slices

**Figure 1:**
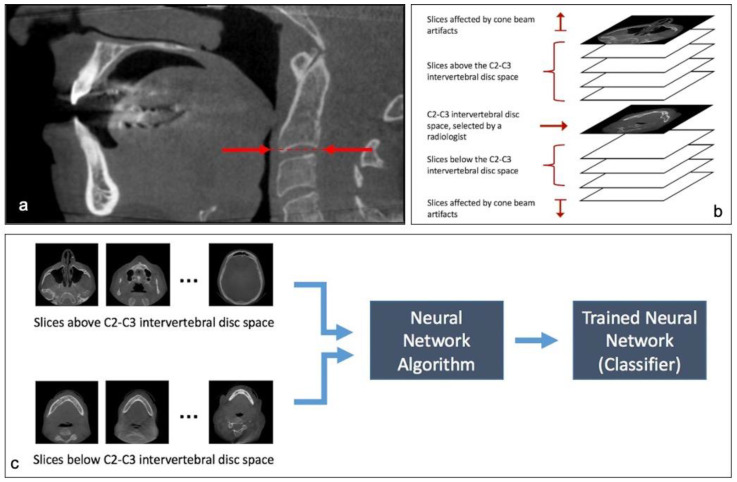
(**a**). The radiologist recorded the midpoint of C2–C3 intervertebral disc in the mid-sagittal view and recorded the axial slice number of this boundary, if any. (**b**). Classification of axial slices into cone beam artifacts and slices above and below the C2–C3 intervertebral disc space. (**c**). Process of training the Classifier neural network with the two groups of data created by the radiologist.

In order to create training data for this neural network, axial slices were divided into two groups: (a) slices at the C2–C3 level and below, and (b) slices above the C2–C3 level. In order to classify axial slices for all CBCT scans in a reproducible fashion, the radiologist selected the midpoint of the C2–C3 intervertebral disc space in a mid-sagittal slice as the boundary between these two groups (Figure 1a).

On average, the first and last 7 slices at both edges of the scan were determined to be cone beam artifacts created by the i-CAT Digital Imaging machine (Imaging Sciences International, Hatfield, PA, USA). These slices were not used for training the neural network (Figure 1b).

From the total 11,252 slices (each slice being a 536 × 536 pixel image), 3222 slices were below the midpoint of C2–C3 intervertebral disc space and 8030 were above, as determined by an expert radiologist. We used the TensorFlow Hub project at https://github.com/tensorflow/hub, accessed on 1 December 2019, to utilize the Inception V3 neural network model that had been trained on the ImageNet dataset and retrained it using our data to be able to classify our dataset into slices that are either above or below the C2–C3 intervertebral disc space (Figure 1c).

The classifier assigns a confidence level between 0% and 100% to each slice. This number represents the confidence level of the classifier that this slice is below the C2–C3 level. For example, a confidence level of 4% means that the classifier is 96% confident that this slice is above the C2–C3 level. For an entire scan composed of many slices, we fed each slice to the classifier independently. We then used linear regression to find a step function from 0% to 100% that most closely matched our data. The slice number represented by this step function was then taken to be the boundary of the C2–C3 level chosen by the neural network.

### 2.2. Step 2: Detection and Localization of Calcifications

**Figure 2:**
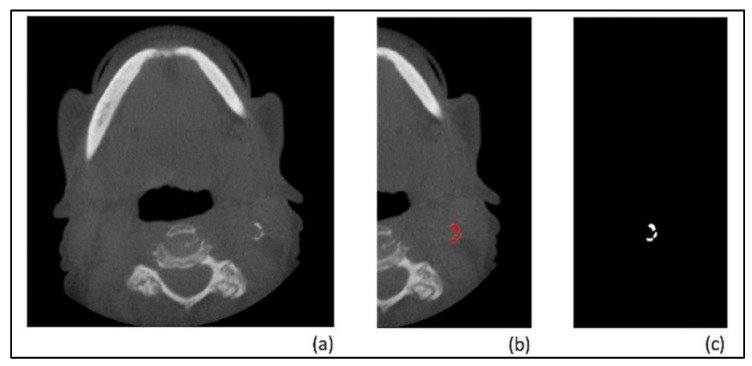
(**a**) an axial slice with a cervical carotid artery calcification. (**b**) the same image after the calcification has been annotated by the radiologist for training. (**c**) the black and white mask. (**b**,**c**) above are cropped horizontally for demonstration purposes—the actual masks have the same dimensions as the original image.

The goal of this step was to train a second neural network capable of detecting and localizing carotid artery calcifications in the axial slices from the output of step 1. Out of our 3222 axial slices below the C2–C3 level, 225 of them contained carotid artery calcifications. The expert radiologist annotated these slices by drawing red pixels over the calcification occurrences. Then a computer program converted the resulting images into black and white masks where white pixels represented calcification occurrences and black pixels represented everything else, i.e., bone, soft tissue, air, etc. (Figure 2).

Image segmentation refers to labeling certain regions of the region of interest on the input image. Semantic segmentation is a technique used for image segmentation. In this technique, each pixel in the input image is assigned a label. In the present study, the labels for each pixel are: calcification region and background. Essentially, the goal of pixel-level segmentation, is to predict the group of pixels which represent the calcification region in the CBCT scan. The following approaches were implemented for image segmentation for the detection and localization of the CAC instances. 

#### 2.2.1. Method A

**Figure 3:**
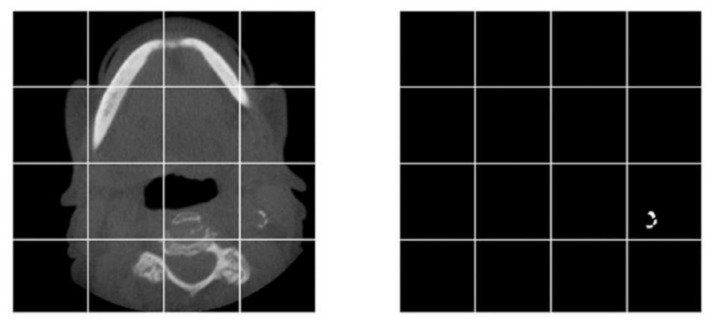
A sample of dividing an image and its corresponding mask into smaller pieces for training the neural network.

Since the number of positive pixels for calcification were significantly smaller than the total number of pixels in one axial slice, each image and its corresponding mask were divided into random patches of size 192 × 192 pixels for training the segmentation neural network. The patches were generated at random coordinates of the original image, and overlapping patches were allowed (Figure 3).

**Figure 4:**
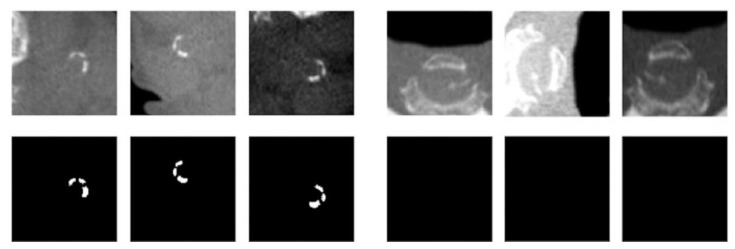
A sample of data augmentation for regions with and without calcification (**first row**) and their corresponding masks (**second row**).

Data augmentation techniques were used to generate new data from the existing data set. Data augmentation also enabled us to prevent overfitting the trained model, thereby allowing the model to be able to detect anatomical variations beyond the training data set. By applying random rotations, skewing, flips, scaling, and brightness adjustments, we were able to generate approximately 5000 variations for each original image (Figure 4).

**Figure 5:**
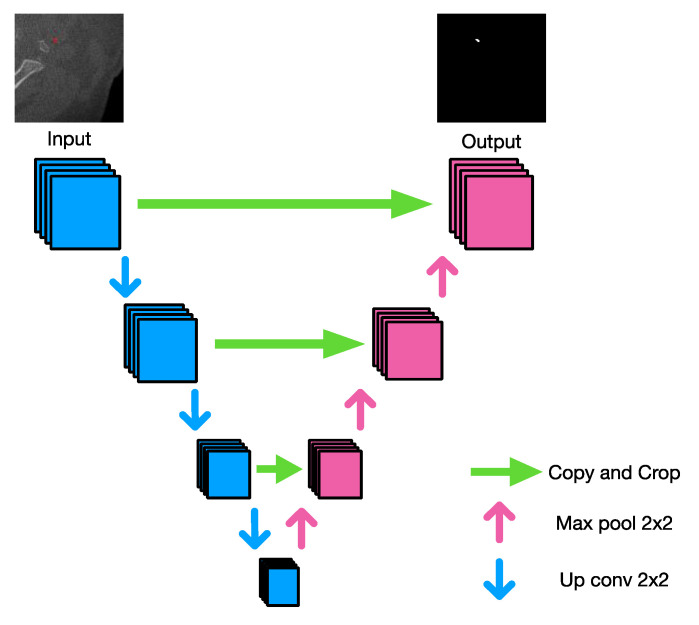
U-Net architecture.

U-net is a convolutional neural network proposed for semantic segmentation. It has been used for performing segmentation in many medical applications. The architecture is presented in Figure 5. 

The U-shaped architecture helps the model to capture both local as well as global features. In our case, as the object is extremely small, the local features help in understanding the underlying structure of the calcification region in higher spaces which might not even be visible to the naked eye. As discussed in the upcoming sections, the base model for both proposed methods is the same. In Method B, we have proposed a variation of training the baseline model proposed in Method A to further boost the performance of the proposed model.

**Figure 6:**
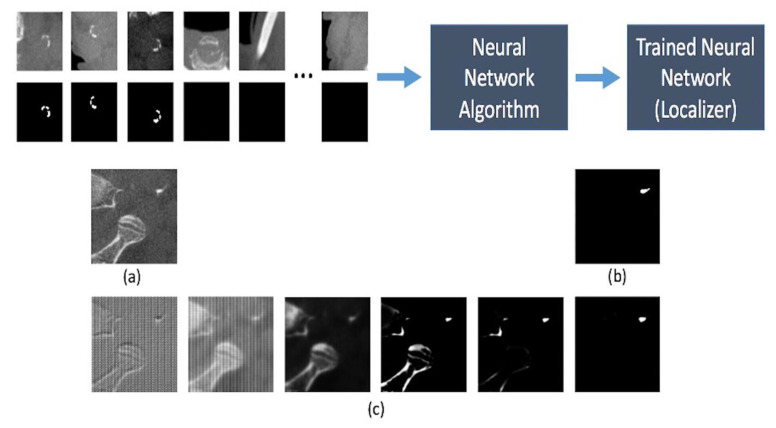
A sample of how the neural network iteratively trains itself. (**a**) an image that the neural network is trying to learn how to analyze. (**b**) the mask created by the radiologist. (**c**) from left to right, demonstrates the neural network getting more accurate in localizing the calcification after being trained on progressively more data.

We used the U-Net neural network architecture to implement a neural network capable of segmenting the image and finding calcification instances. The code for U-Net is available at https://github.com/milesial/Pytorch-UNet, accessed on 1 February 2020. Our implementation segmented pixels of the images in the augmented data set into one of two classes: (1) With CAC (carotid artery calcification) and (2) Without CAC (e.g., bone, soft tissue, air) (Figure 6).

#### 2.2.2. Method B

The aim of this method was to further improve the semantic segmentation performance of the system and better classify each pixel as having calcification or not. To perform this, we extracted two patches of 224 × 224 pixels at fixed locations from the center of the image. Each patch was then fed into U-Net for the semantic segmentation task.

**Figure 7:**
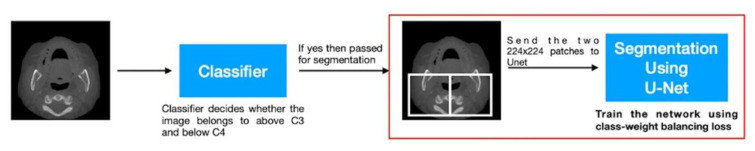
Presents the proposed end-to-end pipeline for calcification detection using the classifier described in Step 1 and the segmentation network described in Method B.

The per-pixel classification problem in this case is extremely challenging due to the issue of class imbalance. In certain patches, there are no calcification regions, and in the ones which do contain a calcification region, the number of such pixels is extremely low. Thus, to overcome this challenge, the U-Net network was trained using the class-weighted loss cross-entropy loss function. For every wrong classification of the calcification pixel, more penalty was added to the loss value (Figure 7).

Ultimately, the three models implemented for image segmentation are as follows:

1. Classifier + U-Net + Multi-patch: this is the model presented as Method A and acts as the baseline model.
2. Classifier + U-Net + 2-patch: this is the model presented as first improvement from Method-A. In this case we have added broad supervision for localizing the calcification areas on patch-level.
3. Classifier + U-Net + 2-patch + class weight balancing loss: this is the final improvement compared to the previous models, as in this case we have changed the loss function used to train the U-Net architecture, thereby making the architecture more robust to class-imbalance.

In summary, there are three major components of any end-to-end deep learning framework: data preparation, deep learning architecture design and loss function used to train the architecture. Following are the differences between Method A and Method B:

1. Data preparation: For Method A the input to U-Net is a cropped region of 192 × 192 of the original scanned image. These patches are randomly generated and overlap between patches is allowed. However, for Method B, we crop the image from specific regions. The goal is to use human level supervision to broadly localize the calcification regions, and feed cropped regions from these localized areas as input to the network.
2. Loss function: One of the major challenges of segmenting out the calcification region is that the calcification region is extremely small compared to the full size of the CBCT scan. Thus, implying that, most pixels in the input are labelled as background or non-calcification regions. This creates a class-imbalance problem while training the network.
  To overcome this, in Method-B we used class-weighted loss function. Essentially, we increased the penalty that is put on the network parameters for predicting the calcification region incorrectly compared to incorrectly predicting the non-calcification region. To compare the results, we used U-Nets trained in Method-A with general cross-entropy loss as baselines.

The performance of the CNN model was assessed using the following metrics: sensitivity, specificity, positive predictive value (PPV), negative predictive value (NNV) and diagnostic accuracy are reported. Furthermore, the performance of the segmentation models for methods A and B are presented as mean intersection over union (IoU).

## 3. Results

**Figure 8:**
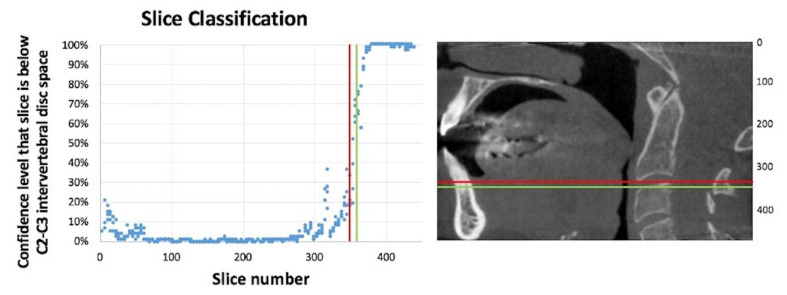
This graph shows the slice number selected by the neural network compared to the slice number selected by the radiologist as the C2–C3 intervertebral disc space for a complete test DICOM study that was not part of the training. The red line (slice 345) was selected by the radiologist, and the green line (slice 357) was selected by the neural network using linear regression. The results for all DICOM studies in the test set were within 15 slices of the correct value.

The performance of the Inception V3 network for the classification of axial slices to above and below C2–C3 levels is presented in Figure 8. The results for all DICOM studies in the test set were within 15 slices of the correct value. For the U-Net CNN, we used 16 CBCT studies (4005 axial slices) that were not part of the training set to test the neural network. Half of the studies had carotid artery calcification (CAC) and half did not. Our approach in method A resulted in 94.2% sensitivity and 96.5% specificity. The positive predictive value is 56.9% and the negative predictive value is 99.7%. The diagnostic accuracy is 96.35% (Table 1).

To test the performance of the segmentation models for methods A and B, we used the m-IoU (mean intersection over union) measure. The following table presents the results obtained with various settings for each method. It can be inferred from the table that adding class-weights balancing loss improves the performance of the network significantly (Table 2).

## 4. Discussion

Numerous advantages have been reported for the application of automated detection algorithms, particularly deep learning, for the analysis of CBCT studies. Conventional radiologic practice involves visual qualitative assessment of radiographic examinations for the detection, characterization and monitoring of disease by a trained physician/dentist. CNN architectures, however, have the capability of recognizing complex radiographic features to provide a quantitative and reproducible assessment of radiographic characteristics in the complex CBCT environment [9]. This is the first study to apply deep learning for the detection of CACs in CBCT images. We trained two models, one for slice classification and one for image segmentation. To our knowledge, this is the first study to apply this approach to 3D cone beam CT data. In this study, we tested the diagnostic performance of two different methods for automated detection of cervical carotid artery calcification. The clinician’s evaluation of presence or absence of CAC in the CBCT scans served as the gold standard [26]. We initially trained the Inception V3 neural network to classify the dataset into slices above and below the C2-C3 intervertebral disc space to limit the field of view to the anticipated location for cervical carotid artery calcification. Yadav and colleagues have previously employed the Inception V3 DCNN model to classify cervical lymphadenopathy [27]. This model has also been trained to detect breast cancer on histopathology images [28]. 

This study implemented the U-Net network to develop the three models presented in Table 2. U-Net is one of the most widely used neural network architectures for biomedical image segmentation [29]. The following models were implemented to train the CNN architecture: Classifier + U-Net + Multi-patch, Classifier + U-Net + 2-patch, Classifier + U-Net + 2-patch + class weight balancing loss. MIoU values of the above three models presented in the results in Table 2 showcase incremental improvement from going from Method A to Method B. It can be observed from the table that each modification improved the result by 4.5% at first and finally by 8.2% compared to the baseline model which is Method A.

In our segmentation pipeline, we provide 3 different methods, however, the difference lies in generating the data and the loss function. Since the base model, i.e., the U-Net architecture is common between all three methods, the models perform at par in terms of computation complexity such as memory consumption and inference time.

Method A rendered excellent diagnostic accuracy of 96.35% with sensitivity and specificity rates of 94.2% and 96.5%, respectively. Method B improved on Method A by focusing the semantic segmentation (i.e., classification of each pixel as having calcification or not) on specific fixed areas of the sagittal images and using class weight balancing loss to yield an improved performance with Mean IoU of 0.83. In method A the patches are generated randomly and in method B the patches are generated in fixed places. The reason both methods are presented in the paper is to showcase that our AI pipeline will work with good performance even if the model does not know the exact area in which the calcification might occur, thus making the method generalizable. There are trade-offs between Method A and Method B in terms of generalization and accuracy. By generalization the authors are referring to obtaining scans in which the position of the calcification region is not known prior to modeling. Thus, both the methods are important.

Numerous studies have investigated the applications of AI in dentistry including dental charting, identification and characterization of anatomy, caries detection and classification and diagnosis of pathology on various imaging modalities [30,31,32,33,34,35,36]. Few articles have applied deep learning techniques for CAC detection in various imaging modalities. Kats and colleagues utilized the Faster Region-based CNN for the detection of CAC on panoramic radiographs. The diagnostic accuracy of their network was 83% with sensitivity and specificity of 75% and 80% respectively [24]. In another study in 2019, Lindsey and Garami developed a 50-layer CNN to classify carotid artery stenosis on ultrasonography images with performance accuracy of 98.3% [25]. In a more recent study in 2021, Bortsova and colleagues trained an ensemble network comprised of four deep learning models for automatic segmentation and quantification of intracranial carotid artery calcifications in multi-detector computed tomography scans (MDCT). The network was trained on 2319 scans and validated and tested on 230 scans and the segmentation performance of the AI model was evaluated qualitatively by visual assessment of the segmentation in comparison with the manual segmentation by blind expert observers. Their results demonstrated an acceptable performance for the AI model with a sensitivity of 83.8% and PPV of 88% [23]. The dataset in the present study most closely resembles the dataset in the latter study. While the overall diagnostic accuracy of our system was high (96.3%), the positive predictive value in the present study was notably low (56.9%), likely due to significantly lower total number of slices with CAC relative to those without CAC which may be improved by limiting the region of interest to the anticipated location for CAC based on the adjacent anatomical landmarks including the hyoid bone and the cervical vertebrae. Another study by Xu et al. applied a deep learning network, VWISegNet, to segment atherosclerotic plaque in magnetic resonance vessel wall imaging. Their model achieved a Dice Similarity Coefficient (DSC) of 93.8% for lumen contours and 86% for outer wall contours which was higher relative to the traditional U-Net, Attention U-Net and Inception U-Net [37].

This study presents with several limitations. The single source and homogenous dataset obtained from one CBCT machine (iCAT Digital Imaging) impacts the generalizability of the results. Furthermore, no power analysis was performed to determine the required sample size for model development. As a pilot study we had a limited sample size with no validation dataset. Therefore, to improve the performance of the algorithm, we must include more heterogenous data from different institutions and machines with different acquisition parameters. Techniques such as cross-validation of training and test data can help validate the approach further. Lastly, our dataset was limited to studies with CAC, and other neck calcifications were excluded. Inclusion of other calcifications enhances the clinical value of the deep learning model in assisting the clinician in differentiating between the different calcifications and determining those that require medical follow up. 

In conclusion, it can be inferred that a reliable deep learning model can be trained to detect vascular calcifications in 3D dental imaging which can serve as a valuable tool for dental healthcare providers who are not familiar with soft tissue anatomy of the neck in 3D imaging. Furthermore, modifying the input image and changing the loss function, from Method A to Method B, improves the semantic segmentation tasks by 8.2%. After conducting this pilot study, we strongly believe that the growing field of AI can help radiologists be more efficient in keeping up with the ever-increasing number of scans.

## Figures and Tables

**Table 1:** Distribution of slices with and without CAC and those detected and not detected by the neural network.

	Slices with CAC	Slices without CAC
Total number of slices	189	3816
Neural network detected CAC	178 (TP)	135 (FP)
Neural network did not detect CAC	11 (FN)	3681 (TN)

**Table 2:** Performance of the neural network using various settings reported as mean intersection over union.

Model	Mean IoU
Classifier + U-Net + Multi-patch (Method A)	0.7626
Classifier + U-Net + 2-patch	0.7975
Classifier + U-Net + 2-patch + class weight balancing loss (Method B)	0.8251

## Data Availability

This is an ongoing project and data are utilized for the next phase and cannot be shared openly at this time.
